# Complete Genome Sequence of a Novel RNA Virus Identified from a Deep-Sea Animal, *Osedax japonicus*

**DOI:** 10.1264/jsme2.ME18089

**Published:** 2018-10-13

**Authors:** Syun-ichi Urayama, Yoshihiro Takaki, Takuro Nunoura, Norio Miyamoto

**Affiliations:** 1 Research and Development Center for Marine Biosciences, Japan Agency for Marine-Earth Science and Technology (JAMSTEC) 2–15 Natsushima-cho, Yokosuka, Kanagawa 237–0061 Japan; 2 Department of Subsurface Geobiological Analysis and Research, JAMSTEC 2–15 Natsushima-cho, Yokosuka, Kanagawa 237–0061 Japan; 3 Laboratory of Fungal Interaction and Molecular Biology (donated by IFO), Department of Life and Environmental Sciences, University of Tsukuba Tsukuba, Ibaraki, 305–8577 Japan

**Keywords:** RNA virus, deep-sea animal, dsRNA

## Abstract

The deep sea, the largest biosphere on Earth, nurtures a large variety of animals. However, no virus that infects deep-sea animals has been found. We herein report the first full-length RNA viral genome sequence identified from the deep-sea animal, *Osedax japonicus*, called Osedax japonicus RNA virus 1 (OjRV1). This sequence showed the highest amino acid sequence similarity to a virus of the family *Togaviridae*. However, the phylogenetic position and genome structure of OjRV1 differed from those of viruses in *Togaviridae*. These results suggest that OjRV1 belongs to a new virus family and that deep-sea animals may associate with new viruses.

A virus is a universal genetic element that affects the physiology and evolution of life. Historically, virus discovery started with the identification of “filterable” animal and plant pathogens around 1900. Over the next 100 years, scientists found numerous viruses as causal agents for various diseases, and “illnesses” of organisms were generally required to inspire a motive for virus surveillance (17). Therefore, in the case of animal viruses, knowledge on their diversity is limited to findings derived from terrestrial and shallow water animals that are of economical and epidemiological importance. However, diverse viral sequences were also recently detected from animals in which no clear disease symptoms were observed (19). This finding indicates the presence of many unrecognized viruses in animals and the importance of the virus surveillance in a phenotype-independent manner (19) of diverse organisms and/or habitats to understand the viral diversity associated with animals.

The deep sea is the largest biosphere on Earth, and it nurtures a large variety of animals (4). The identification of deep-sea animal viruses will provide important information on animal virus diversity and lineages by filling in the missing pieces of their phylogenies. However, to the best of our knowledge, no virus that infects deep-sea animals has been reported to date, most likely because of the difficulties associated with identifying viral diseases in deep-sea animals without rearing. The establishment of breeding methods for various deep-sea animals will contribute not only to the discovery of viruses, but also to our understanding of the properties of virus particles and the influence of viruses on the host phenotype.

Bone-eating worms of the genus *Osedax* are marine invertebrates that belong to the phylum *Annelida*, family *Siboglinidae* (18). *Osedax* exclusively inhabit sunken whale bones under natural conditions and may colonize the bones of other vertebrate species under experimental conditions (7). *Osedax* lack a digestive tract, including a mouth, gut, and anus. They appear to use their root as their digestive organ. Gene expression in the root epidermis supports *Osedax* degrading vertebrate bones and absorbing nutrients inside the bones from the root (13). *Osedax* females harbor heterotrophic bacteria in their bacteriocytes, which are located inside the root. These endosymbionts are infected from the environment during metamorphosis (12).

dsRNA is a hallmark of RNA virus infection (15), and is used for phenotype-independent RNA virus surveillance. To detect RNA viruses, dsRNA is purified using cellulose resin and analyzed (16, 22). A novel dsRNA sequencing method, called fragmented and loop-adapter-ligated dsRNA sequencing (FLDS), has recently enabled us to efficiently elucidate RNA virus genome(s) (23). This technique was originally applied to the marine diatom community (23) and functioned for the viral surveillance of an insect (9). In the present study, we report the complete nucleotide sequence of a novel RNA virus identified from the deep-sea animal, *O. japonicus*, using FLDS.

We collected *O. japonicus* specimens from whale bones obtained at a depth of 226 m off Cape Noma Misaki via a remotely operated vehicle, *Hyper-Dolphin*, on the research vessel (R/V) *Natsushima* on March 28 (NT12-07) and April 13 (NT12-09), 2012. *O. japonicus* were kept in 100-L tanks at 11°C in the laboratory (12). *Neptunomonas japonica*, a species of symbiotic bacteria of *O. japonicus*, was cultured with marine broth 2216 (MB; Difco, Sparks, MD, USA) at 20°C (14).

The purification and sequencing of dsRNA were performed as previously described (23). In brief, to detect viral dsRNA, total nucleic acids were extracted from the females of *O. japonicus* with BioMasher II (Nippi, Tokyo, Japan) in dsRNA extraction buffer and phenol-chloroform. dsRNA was purified with cellulose resin and then subjected to agarose gel electrophoresis (Fig. 1). After the further purification of dsRNA, dsRNA genome segments were converted to cDNA by the FLDS method (23). In high throughput sequencing, we used IonPGM (Thermo Fisher Scientific, Waltham, MA, USA). Regions with low sequence read coverage (<4-fold) in contigs were sequenced using a 3730xl DNA analyzer (Thermo Fisher Scientific) with the RT-PCR products as templates. Raw sequence reads were processed with the CLC Genomics Workbench (CLC Bio, Aarhus, Denmark) as described previously (23). Contigs were obtained *de novo* exclusively with the CLC Genomics Workbench (CLC Bio) using trimmed reads and Sanger sequencing reads with the following parameters: a minimum contig length of 500, word value set to Auto, and bubble size set to Auto. The major two contigs were manually examined and extended using the Tablet viewer (11) and Genetyx software version 13.1.0 (Genetyx Corp., Tokyo, Japan). To identify the origins of the NGS reads, processed reads were mapped on the OjRV1 genome with the CLC Genomics Workbench using the following parameters: mismatch cost of 2, insertion/deletion cost of 3, length fraction of 0.8, and similarity fraction of 0.8. The mapped reads on the OjRV1 genome were counted. Unmapped reads were applied to a homology search using Blastn and Blastx with the NCBI non-redundant nucleotide (Nt) database and NCBI non-redundant protein (Nr) database, respectively, and assigned by MEGAN ver. 6.6.1 (6).

In the phylogenetic analysis, multiple alignments based on the deduced amino acid sequences of putative RNA-dependent RNA polymerase (RdRp) or the helicase domain in the contigs were constructed by MUSCLE (5) in MEGA5 (21). The alignment was further trimmed by trimAl (option: -gt 1) (3) to remove all gaps in the alignment in order to avoid the overestimation of informative gaps. Phylogenetic analyses were conducted using RAxML ver. 8.2.7 (20) with the model of amino acid substitution selected by ProtTest2.4 (1) and visualized using FigTree ver. 1.4.2 (http://tree.bio.ed.ac.uk/software/figtree/).

Two dsRNA bands were detected from *O. japonicus* by gel electrophoresis (Fig. 1A) and were defined as RNA-1 (~15 kb) and RNA-2 (~8 kb). As a result of sequencing and assembly, we obtained the complete nucleotide sequences of RNA-1 (GenBank accession LC367326) and RNA-2 (GenBank accession LC367327), which consisted of 13,170 and 7,176 bp, respectively (Fig. 1B). The origins of the NGS reads are summarized in Table S1. The 5′- and 3′-terminal sequences of the sense strands of the two RNAs were similar to each other (Fig. 1C). The two RNAs had similar genome GC contents (49.1 and 49.9%). A single open reading frame (ORF) was identified in each RNA sequence. A conserved domain search (10) revealed that the predicted amino acid sequence of ORF1 (an ORF on RNA-1) contained conserved motifs of viral RdRp (pfam00978, RdRp_2, e-value=7.26e-54) and viral RNA helicase (pfam01443, Viral_helicase1, e-value= 6.33e-12). The predicted amino acid sequence of ORF2 (an ORF on RNA-2) contained conserved motifs of an alphavirus E1 glycoprotein (pfam01589, alphavirus E1 glycoprotein, e-value=5.18e-25). These results suggested that RNA-1 and RNA-2 were genomic segments of an RNA virus and most likely derived from a single RNA virus. We did not detect the dsRNA bands of RNA-1 and RNA-2 from a cultured stock of *N. japonica*, which is a species of *O. japonicus* symbiont, using an *O. japonicus* culture (data not shown). Therefore, we called this novel bisegmented virus Osedax japonicus RNA virus 1 (OjRV1).

The known sequences that showed the lowest e-values in the BLASTp analysis (2) against the Nr database for ORF1 and ORF2 were the nsP4 protein of *Ross River virus* (*Togaviridae*, e-value=1e-55) and the structural polyprotein of *Venezuelan equine encephalitis virus* (*Togaviridae*, e-value= 7e-20), respectively. The results of the BLASTp search are summarized in Table S2. These viruses belong to the family *Togaviridae*, and have a non-segmented positive sense ssRNA genome (10–12 k nucleotides) with a poly(A)-tail (8). This monopartite virus genome contained two ORFs (Fig. S1). The 5′-proximal ORF encodes a replication protein. The 3′-proximal ORF encodes the nucleocapsid protein and two viral glycoproteins, and these proteins are translated from a subgenomic mRNA, which is colinear with the 3′ one-third of the genome. The genetic organization of OjRV1 in RNA 1 and 2 was similar to the 5′ and 3′ sides, respectively, of the non-segmented genome in *Togaviridae*. On the other hand, the OjRV1 genome had no poly(A)-tail and was longer (20,346 nucleotides in length) than the known viruses in *Togaviridae* (9.7–11.8 kb). The RdRp and helicase domains of OjRV1 presented similarities with those in *Closteroviridae*, *Virgaviridae*, *Bromoviridae*, and *Idaeovirus* in addition to *Togaviridae*. The phylogenetic analysis based on the amino acid sequences of RdRp and helicase domains confirmed that *Togaviridae* was the closest known viral family, but was phylogenetically distinct from OjRV1 (Fig. 2). Five genera (*Crinivirus*, *Furovirus*, *Pecluvirus*, *Tobravirus*, and *Idaeovirus*) in these virus groups include viruses with bisegmented genomes; however, these viruses contain three or more ORFs. These results suggest that OjRV1 is a novel bipartite RNA virus that belongs to a new virus family.

We were unable to verify whether OjRV1 replicated in *O. japonicus* cells because *O. japonicus* harbors endosymbionts and the rearing conditions were not aseptic. However, we assumed that the biomass of these associated (micro)organisms was too small to possess dsRNA visible in gel electrophoresis. In addition, RdRp identified in the present study has similarities to viruses that infect eukaryotes, and most eukaryotic reads contained in the FLDS library were derived from *Osedax* (Table S1). These results suggest that the RNA virus identified in the present study most likely infected *O. japonicus*. To confirm the infection and influence of this virus on the host phenotype, the detection of intracellular viral RNA by FISH technology is necessary. However, it was impossible to conduct a FISH analysis in the present study because the *Osedax* strain was lost after the experiment and, as a consequence, we lost the virus. The effect of this virus on this loss has not yet been clarified.

Deep-sea animals may associate with new viruses that differ from those we have studied to date. A virus similar to OjRV1 has not yet been obtained in large-scale shotgun RNA-seq targeting non-deep sea invertebrates (19). To date, most of the deep-sea viruses identified have been from floating virus particles or microorganisms; however, in the present study, we demonstrated the potential of deep-sea animals as a novel virus source. We consider this research to be of prime importance in virus research on deep-sea animals.

## Supplementary Material



## Figures and Tables

**Fig. 1:**
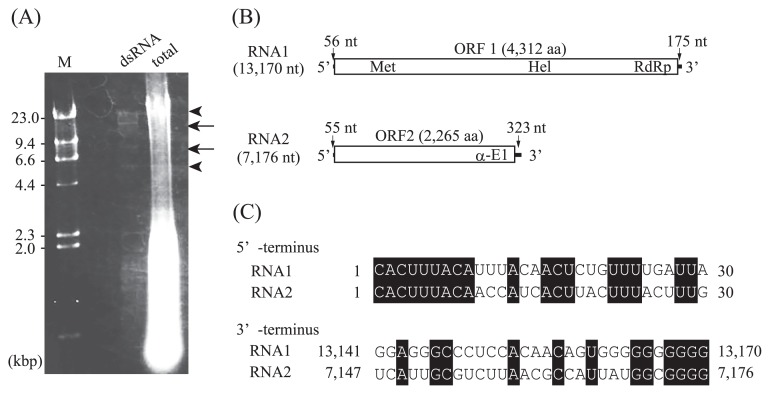
Characterization of dsRNA molecules detected from *Osedax japonicus*. (A) Agarose gel (1%) electrophoresis of the total nucleic acids and dsRNA of *O. japonicus*. Arrows indicate dsRNA bands (RNA 1 and RNA 2) identified by FLDS. Arrowheads show non-dsRNA bands, which were not detected in other gel analyses. Lane M, *Hin*dIII-digested lambda DNA marker. (B) Predicted ORFs and identified domains: Met, Vmethyltransf super family; Hel, Viral_helicase1 super family; RdRp, RdRP_2 super family; α-E1, Alpha_E1_glycop super family. (C) Multiple alignments of the 5′- and 3′-terminal regions of the coding strands of RNA-1 and RNA-2.

**Fig. 2:**
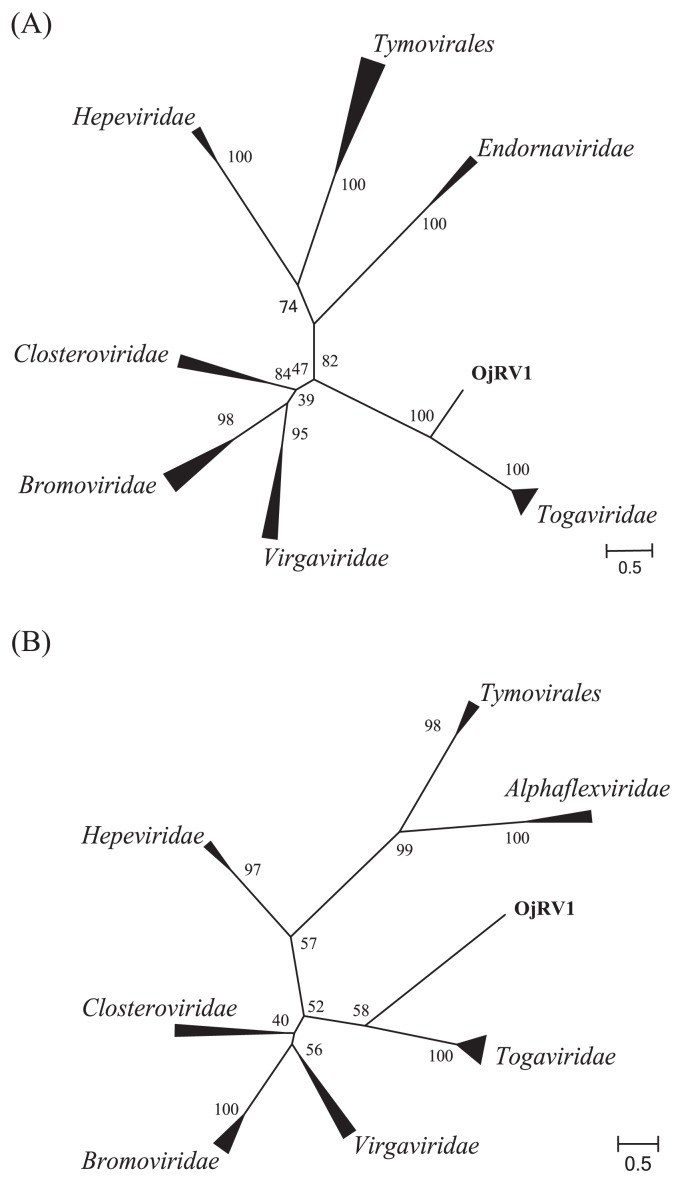
Maximum likelihood trees of (A) RdRp and (B) helicase domain amino acid sequences of OjRV1 and related ssRNA viruses. The numbers indicate the percentage bootstrap support from 1,000 RAxML bootstrap replicates. The accession numbers and full virus names are listed in Table S3 and S4. The scale bar represents substitutions per site.
