# Supplementary material for: Complete Genome Sequence of a Novel RNA Virus Identified from a Deep-Sea Animal, Osedax japonicus
Source: Microbes Environ. 2018 Oct 13;33(4):446–9. doi: 10.1264/jsme2.ME18089 (PMC6308001; doi:10.1264/jsme2.ME18089)
Supplement: Supplementary file 1 [file 33_446_s1.pdf]

Fig S1: Genome organization of known viruses in *Togaviridae*

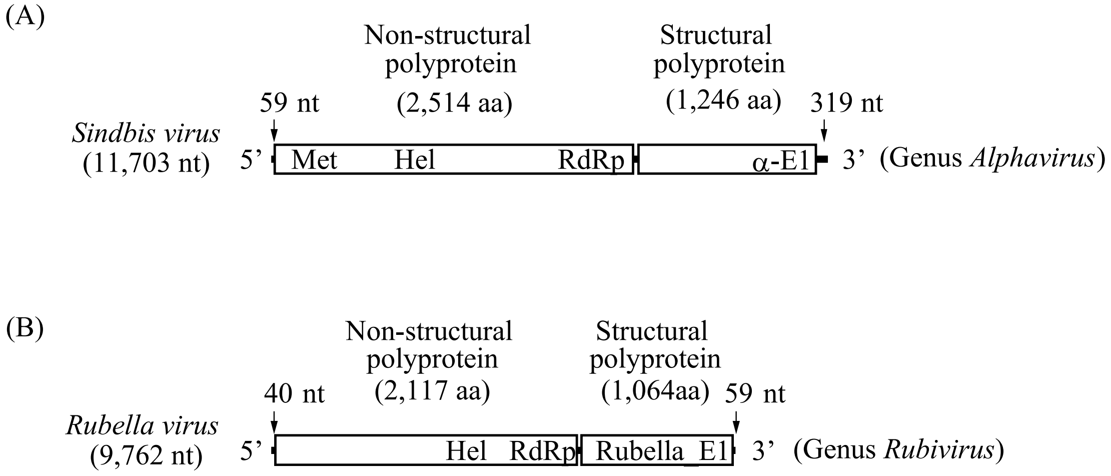

Genome organization of known viruses in *Togaviridae*; (A) *Sindbis virus* and (B) *Rubella virus*. Domains: Met, Vmethyltransf super family; Hel, Viral_helicase1 super family; RdRp, RdRP_2 super family; α-E1, Alpha_E1_glycop super family; Rubella E1, Rubella membrane glycoprotein E1.

Table S1: Origins of the FLDS reads

	reads	ratio (%)
trimmed reads	134513	100.0
OjRV	125004[*]	92.9
Cell	1001[**]	0.7
Eukcariota	749	
Osedax	153	
Symbiodinium	35	
Spironucleus	15	
Others	546	
Bacteria	246	
Not assigned	6	
Not assigned	59[**]	0.0
No hit	8449[**]	6.2

[*]: Count of mapped reads on the OjRV genome sequence.

**: Homology search was performed using Blastn and Blastx, and the results were assigned by MEGAN (6).

Table S2: Blastp hit list of predicted ORF1.

Database	virus	protein	accession	e-value	family
non-redundant protein sequences	Ross River virus	nsP4 protein	NP_740681	2.00E-55	Togaviridae
non-redundant protein sequences	Getah virus	nonstructural polyprotein	ARK36627	2.00E-50	Togaviridae
non-redundant protein sequences	Sagiyama virus	polyprotein	BAA92845	2.00E-50	Togaviridae
non-redundant protein sequences	Alphavirus M1	nsp1234	ABK32031	3.00E-50	Togaviridae
non-redundant protein sequences	Mayaro virus	Nsp4	ALI88625	8.00E-50	Togaviridae
non-redundant protein sequences	Middelburg virus	nonstructural polyprotein	AAA96653	1.00E-49	Togaviridae
non-redundant protein sequences	Semliki forest virus	putative RNA-dependent RNA polymerase	CAA75053	2.00E-49	Togaviridae
non-redundant protein sequences	Chikungunya virus	non-structural polyprotein	ADZ04935	2.00E-48	Togaviridae
non-redundant protein sequences	Bebaru virus	non-structural polyprotein precursor nsP1234	YP_008901140	2.00E-48	Togaviridae
non-redundant protein sequences	O'nyong-nyong virus	nonstructural protein P4	NP_740706	5.00E-48	Togaviridae
non-redundant protein	Sleeping disease virus	non structural protein P4	NP_740656	5.00E-48	Togaviridae

sequences					
NCBI Protein Reference Sequences	Ross River virus	nsP4 protein	NP_740681	1.00E-55	Togaviridae
NCBI Protein Reference Sequences	Getah virus	nsP1234 polyprotein	YP_164438	9.00E-50	Togaviridae
NCBI Protein Reference Sequences	Semliki Forest virus	nonstructural protein nsP4	NP_740668	1.00E-49	Togaviridae
NCBI Protein Reference Sequences	Mayaro virus	nonstructural protein nsP4	NP_740690	3.00E-49	Togaviridae
NCBI Protein Reference Sequences	Middelburg virus	non-structural polyprotein	YP_009058892	1.00E-48	Togaviridae
NCBI Protein Reference Sequences	Bebaru virus	non-structural polyprotein precursor nsP1234	YP_008901140	1.00E-48	Togaviridae
NCBI Protein Reference Sequences	O'nyong-nyong virus	nonstructural protein P4	NP_740706	3.00E-48	Togaviridae
NCBI Protein Reference Sequences	Sleeping disease virus	non structural protein P4	NP_740656	4.00E-48	Togaviridae
NCBI Protein Reference Sequences	Chikungunya virus	nonstructural polyprotein	NP_690588	3.00E-47	Togaviridae
NCBI Protein Reference Sequences	Ndumu virus	non-structural polyprotein precursor nsP1234	YP_008888544	1.00E-46	Togaviridae
NCBI Protein Reference Sequences	Fort Morgan virus	nsP4	YP_003324594	4.00E-46	Togaviridae
NCBI Protein Reference Sequences	Sindbis virus	nsp4 nonstructural protein	NP_740669	8.00E-46	Togaviridae
NCBI Protein Reference Sequences	Salmon pancreas disease virus	non structural protein P4	NP_740638	4.00E-45	Togaviridae

NCBI Protein Reference Sequences	Tai Forest alphavirus	non-structural polyprotein	YP_009333615	7.00E-45	Togaviridae
NCBI Protein Reference Sequences	Madariaga virus	RNA-directed RNA polymerase nsp4	YP_009020586	4.00E-44	Togaviridae
NCBI Protein Reference Sequences	Barmah Forest virus	non-structural polyprotein precursor nsP1234	NP_597797	4.00E-44	Togaviridae
NCBI Protein Reference Sequences	Whataroa virus	non-structural polyprotein precursor nsP1234	YP_008888546	1.00E-43	Togaviridae
NCBI Protein Reference Sequences	Western equine encephalitis virus	nonstructural protein nsP4	NP_818936	2.00E-43	Togaviridae
NCBI Protein Reference Sequences	Venezuelan equine encephalitis virus	putative nonstructural protein nsP4	NP_740699	6.00E-43	Togaviridae
NCBI Protein Reference Sequences	Southern elephant seal virus	non-structural polyprotein precursor nsP1234	YP_008888545	2.00E-42	Togaviridae
NCBI Protein Reference Sequences	Eilat virus	non-structural polyprotein precursor nsP1234	YP_008901141	3.00E-42	Togaviridae
NCBI Protein Reference Sequences	Eastern equine encephalitis virus	NS4	NP_740652	4.00E-42	Togaviridae
NCBI Protein Reference Sequences	Aura virus	Nonstructural protein nsP4	NP_819013	3.00E-41	Togaviridae
NCBI Protein Reference Sequences	Highlands J virus	nsP4	YP_002802304	2.00E-40	Togaviridae
NCBI Protein Reference Sequences	Potato yellow vein virus	RNA-dependent RNA polymerase	YP_829128	4.00E-18	Closteroviridae
NCBI Protein Reference Sequences	Beihai charybdis crab virus 1	RdRp	YP_009333242	2.00E-15	unclassified
NCBI Protein	Hubei virga-like	RdRp	YP_009337693	3.00E-14	unclassified

Reference Sequences	virus 15				
NCBI Protein Reference Sequences	Olive latent virus 2	2a protein	NP_620043	2.00E-11	Bromoviridae
NCBI Protein Reference Sequences	Grapevine leafroll-associated virus 5	RdRp gene product	YP_004901687	2.00E-11	Closteroviridae
NCBI Protein Reference Sequences	Grapevine leafroll-associated virus 1	POL gene product	YP_004940642	3.00E-11	Closteroviridae
NCBI Protein Reference Sequences	Blueberry virus A	RNA dependent RNA polymerase	YP_006638806	3.00E-11	Closteroviridae
NCBI Protein Reference Sequences	Parietaria mottle virus	p2 protein	YP_006447	8.00E-11	Bromoviridae
NCBI Protein Reference Sequences	Hubei virga-like virus 2	RdRp	YP_009337412	2.00E-10	unclassified
NCBI Protein Reference Sequences	Adelphocoris suturalis virus	ORF1	YP_009336476	4.00E-10	unclassified
NCBI Protein Reference Sequences	Grapevine leafroll-associated virus 13	RNA-dependent RNA polymerase, partial	YP_009241367	9.00E-10	Closteroviridae
NCBI Protein Reference Sequences	Alfalfa mosaic virus	89.7 kd protein	YP_053235	1.00E-09	Bromoviridae
NCBI Protein Reference Sequences	Hubei virga-like virus 9	RdRp	YP_009336553	1.00E-09	unclassified
NCBI Protein Reference Sequences	Tobacco streak virus	putative viral polymerase	NP_620768	1.00E-09	Bromoviridae
NCBI Protein Reference Sequences	Ageratum latent virus	RNA-dependent RNA polymerase	YP_008470970	2.00E-09	Bromoviridae
NCBI Protein Reference	Grapevine leafroll-associated	polyprotein	NP_813795	3.00E-09	Closteroviridae

Sequences	virus 3				
NCBI Protein Reference Sequences	Tulare apple mosaic virus	putative polymerase p2	NP_620754	6.00E-09	Bromoviridae
NCBI Protein Reference Sequences	Citrus leaf rugose virus	RNA-dependent RNA polymerase	NP_613281	1.00E-08	Bromoviridae
NCBI Protein Reference Sequences	Blueberry shock virus	replicase P2	YP_008519305	1.00E-08	Bromoviridae
NCBI Protein Reference Sequences	Grapevine leafroll-associated virus 10	RNA dependent RNA polymerase, partial	YP_002364303	2.00E-08	Closteroviridae
NCBI Protein Reference Sequences	Prunus necrotic ringspot virus	polymerase p2	NP_733824	2.00E-08	Bromoviridae
NCBI Protein Reference Sequences	Brome mosaic virus	RNA-dependent RNA polymerase	NP_041197	3.00E-08	Bromoviridae
NCBI Protein Reference Sequences	Culex negev-like virus 1	RdRp	YP_009388585	3.00E-08	unclassified
NCBI Protein Reference Sequences	Blackberry chlorotic ringspot virus	p2 protein	YP_002308570	5.00E-08	Bromoviridae
NCBI Protein Reference Sequences	Humulus japonicus latent virus	p2 protein	YP_054423	6.00E-08	Bromoviridae
NCBI Protein Reference Sequences	Strawberry necrotic shock virus	RdRp	YP_941472	9.00E-08	Bromoviridae
NCBI Protein Reference Sequences	Privet leaf blotch-associated virus	Replication-associated polyprotein	YP_009305430	2.00E-07	Idaeovirus
NCBI Protein Reference Sequences	Asparagus virus 2	polymerase	YP_002455929	2.00E-07	Bromoviridae
NCBI Protein Reference Sequences	Fragaria chiloensis latent virus	RNA-dependent RNA polymerase	YP_164802	3.00E-07	Bromoviridae

NCBI Protein Reference Sequences	Black currant leaf chlorosis associated virus	nonstructural polyprotein	YP_009361854	8.00E-07	Idaeovirus
NCBI Protein Reference Sequences	Hubei virga-like virus 21	hypothetical protein	YP_009337659	8.00E-07	unclassified
NCBI Protein Reference Sequences	Privet ringsport virus	replication-associated polyprotein 2a	YP_009165997	9.00E-07	Bromoviridae
NCBI Protein Reference Sequences	Elm mottle virus	polymerase	NP_619575	1.00E-06	Bromoviridae
NCBI Protein Reference Sequences	Wuhan heteroptera virus 1	RdRp	YP_009342329	4.00E-06	unclassified
NCBI Protein Reference Sequences	Prune dwarf virus	polymerase P2	YP_611151	4.00E-06	Bromoviridae
NCBI Protein Reference Sequences	Streptocarpus flower break virus	putative replicase	YP_762617	1.00E-05	Virgaviridae

Table S3: Blastp hit list of predicted ORF2.

Database	virus	protein	accession	e-value	family
non-redundant protein sequences	Venezuelan equine encephalitis virus	structural polyprotein	ADA84123	8.00E-20	Togaviridae
non-redundant protein sequences	Eastern equine encephalitis virus	structural polyprotein	ADB08675	2.00E-19	Togaviridae
non-redundant protein sequences	Madariaga virus	structural polyprotein	AHL83773	2.00E-19	Togaviridae
NCBI Protein Reference Sequences	Eastern equine encephalitis virus	E1 protein	NP_740648	3.00E-18	Togaviridae
NCBI Protein Reference	Venezuelan equine encephalitis virus	structural polyprotein	NP_040824	9.00E-18	Togaviridae

Sequences		precursor			
NCBI Protein Reference Sequences	Mayaro virus	envelope glycoprotein E1	NP_740694	2.00E-16	Togaviridae
NCBI Protein Reference Sequences	Aura virus	E1 protein	NP_819019	4.00E-16	Togaviridae
NCBI Protein Reference Sequences	Chikungunya virus	structural polyprotein	NP_690589	6.00E-16	Togaviridae
NCBI Protein Reference Sequences	Whataroa virus	unnamed protein product	YP_005351237	1.00E-14	Togaviridae
NCBI Protein Reference Sequences	Tai Forest alphavirus	structural polyprotein	YP_009333616	2.00E-14	Togaviridae
NCBI Protein Reference Sequences	Sindbis virus	e-1 structural protein	NP_740677	2.00E-14	Togaviridae
NCBI Protein Reference Sequences	Fort Morgan virus	E1 protein	YP_003324599	8.00E-14	Togaviridae
NCBI Protein Reference Sequences	Getah virus	C-P62-6K-E1 polyprotein	YP_164439	2.00E-13	Togaviridae
NCBI Protein Reference Sequences	Bebaru virus	unnamed protein product	YP_005351239	4.00E-13	Togaviridae
NCBI Protein Reference Sequences	Eilat virus	structural protein	YP_006732328	1.00E-10	Togaviridae

Table S4: GenBank/protein accession numbers for the phylogenetic analysis of the RdRp sequences.

Name	Abbrevation	Family/Genus	Interval (aa)	Accession
Chikungunya virus strain S27-African	CHIKV	Togaviridae/ Alphavirus	2,153-2,395	AAN05101

Getah virus from South Korea	GETV	Togaviridae/ Alphavirus	2,146-2,388	AAU85259
Eastern equine encephalitis virus strain FL93-939	EEEV-I	Togaviridae/ Alphavirus	2,176-2,418	ABL84686
O'nyong-nyong virus strain SG650	ONNV	Togaviridae/ Alphavirus	2,192-2,434	AAC97204
Ross River virus strain 9057	RRV	Togaviridae/ Alphavirus	2,160-2,402	ACV66999
Semliki forest virus 42S	SFV	Togaviridae/ Alphavirus	2,107-2,349	CAA27741
Sindbis virus isolate SW6562	SINV	Togaviridae/ Alphavirus	2,166-2,408	AAM10628
Sleeping disease virus	SDV	Togaviridae/ Alphavirus	2,275-2,517	CAC87660
Venezuelan equine encephalitis virus strain 71-180	VEEV-IAB	Togaviridae/ Alphavirus	2,175-2,417	AAC24033
Western equine encephalomyelitis virus strain 71V-1658	WEEV	Togaviridae/ Alphavirus	2,149-2,390	AAF28339
Tobacco streak virus	TSV	Bromoviridae/ Ilarvirus	385-625	AAB48409
Brome mosaic virus	BMV	Bromoviridae/ Bromovirus	387-619	CAA25834
Cowpea chlorotic mottle virus	CCMV	Bromoviridae/ Bromovirus	398-630	NP_613275.1
Peanut stunt virus	PSV	Bromoviridae/ Cucumovirus	426-658	BAA01901
Cucumber mosaic virus	CMV	Bromoviridae/ Cucumovirus	439-671	BAA00263
Broad bean mottle virus	BBMV	Bromoviridae/ Bromovirus	398-630	AAA42741
Tomato aspermy virus	TAV	Bromoviridae/ Cucumovirus	443-675	BAA01514
Tobacco mosaic virus	TMV	Virgaviridae/ Tobamovirus	1,303-1,541	NP_597746
Barley stripe mosaic virus	BSMV	Virgaviridae/ Hordeivirus	449-685	AAA66600
Soil-borne wheat mosaic virus	SBWMV	Virgaviridae/ Furovirus	1,505-1,742	NP_049335
Pea early-browning virus	PEBV	Virgaviridae / Tobravirus	1,433-1,670	CAB37343
Tobacco rattle virus	TRV	Virgaviridae/ -	1,373-1,610	BAA00110
Potato virus X	PVX	Alphaflexiviridae/ Potexvirus	1,161-1,390	P09395
Strawberry mild yellow edge virus	SMYEAV	Alphaflexiviridae/ Potexvirus	1,030-1,258	BAA02082

Shallot virus X	ShVX	Alphaflexiviridae/ Allexivirus	1,339-1,562	AAA47787
Turnip yellow mosaic	TYMV	Tymoviridae/ Tymovirus	1,498-1,720	CAA30322
Eggplant mosaic virus	EPMV	Tymoviridae/ Tymovirus	1,494-1,715	AAA43039
Ononis yellow mosaic virus	OYMV	Tymoviridae/ Tymovirus	1,424-1,645	AAA46796
Kennedya yellow mosaic virus	KYMV	Tymoviridae/ Tymovirus	1,528-1,749	BAA00532
Garlic virus A	GarV-A-JA	Alphaflexiviridae/ Allexivirus	1,257-1,480	NP_569126
Oryza sativa endornavirus	OsEV	Endornaviridae/ Endornavirus	4,240-4,477	YP_438200
Hepatitis E virus	HEV-1	Hepeviridae/ Hepevirus	1,382-1,609	AAA45734
Hepatitis E virus Ct1	HEV-4	Hepeviridae/ Hepevirus	1,396-1,623	Q9IVZ9
Avian hepatitis E virus	aHEV-1	Hepeviridae/ -	1,222-1,450	CAQ16027
Vicia faba endornavirus	VfEV-447	Endornaviridae/ Endornavirus	5,396-5,632	YP_438201
Phytophthora endornavirus 1	PEV1-OR	Endornaviridae/ Endornavirus	4,280-4,517	YP_241110
Beet yellows virus	BYV	Closteroviridae/ Closterovirus	2,720-2,956	AAC25115
Mint virus 1	MV1	Closteroviridae/ Closterovirus	110-347	YP_224091
Plum bark necrosis stem pitting-associated virus	PBNSTaV	Closteroviridae/ Ampelovirus	190-427	YP_001552324
Little cherry virus 2	LChV-2	Closteroviridae/ Ampelovirus	1,831-2,068	NP_891562

Table S5: GenBank/protein accession numbers for the phylogenetic analysis of the Helicase sequences.

Name	Abbrevation	Family/Genus	Interval (aa)	Accession
Chikungunya virus strain S27-African	CHIKV	Togaviridae/ Alphavirus	720-959	AAN05101
Getah virus from South Korea	GETV	Togaviridae/ Alphavirus	719-958	AAU85259
Eastern equine encephalitis virus strain FL93-939	EEEV-I	Togaviridae/ Alphavirus	718-956	ABL84686

O'nyong-nyong virus strain SG650	ONNV	Togaviridae/ Alphavirus	720-959	AAC97204
Ross River virus strain 9057	RRV	Togaviridae/ Alphavirus	719-958	ACV66999
Semliki forest virus 42S	SFV	Togaviridae/ Alphavirus	722-961	CAA27741
Sindbis virus	SINV	Togaviridae/ Alphavirus	725-967	NP_062889
Sleeping disease virus	SDV	Togaviridae/ Alphavirus	749-988	CAC87660
Venezuelan equine encephalitis virus strain 71-180	VEEV-IAB	Togaviridae/ Alphavirus	720-946	AAC24033
Western equine encephalomyelitis virus strain 71V-1658	WEEV	Togaviridae/ Alphavirus	718-956	AAF28339
Brome mosaic virus	BMV	Bromoviridae/ Bromovirus	684-946	NP_041196
Cowpea chlorotic mottle virus	CCMV	Bromoviridae/ Bromovirus	681-943	NP_613278
Peanut stunt virus	PSV	Bromoviridae/ Cucumovirus	721-984	P28726
Cucumber mosaic virus	CMV	Bromoviridae/ Cucumovirus	713-976	NP_049323
Broad bean mottle virus	BBMV	Bromoviridae/ Bromovirus	689-951	NP_659000
Tomato aspermy virus	TAV	Bromoviridae/ Cucumovirus	713-976	NP_620760
Tobacco mosaic virus	TMV	Virgaviridae/ Tobamovirus	832-1,084	NP_597746
Barley stripe mosaic virus	BSMV	Virgaviridae/ Hordeivirus	837-1,109	NP_604474
Soil-borne wheat mosaic virus	SBWMV	Virgaviridae/ Furovirus	1,026-1,288	NP_049335
Pea early-browning virus	PEBV	Virgaviridae / Tobravirus	962-1,214	CAB37343
Tobacco rattle virus	TRV	Virgaviridae/ -	903-1,155	BAA00110
Potato virus X	PVX	Alphaflexiviridae/ Potexvirus	734-963	P09395
Strawberry mild yellow edge virus	SMYEAV	Alphaflexiviridae/ Potexvirus	604-833	BAA02082
Shallot virus X	ShVX	Alphaflexiviridae/ Allexivirus	914-1,142	AAA47787
Turnip yellow mosaic	TYMV	Tymoviridae/ Tymovirus	975-1,204	CAA30322
Eggplant mosaic virus	EPMV	Tymoviridae/ Tymovirus	964-1,192	AAA43039

Ononis yellow mosaic virus	OYMV	Tymoviridae/ Tymovirus	989-1,127	AAA46796
Kennedya yellow mosaic virus	KYMV	Tymoviridae/ Tymovirus	1,001-1,234	BAA00532
Garlic virus A	GarV-A-JA	Alphaflexiviridae/ Allexivirus	831-1,059	NP_569126
Hepatitis E virus	HEV-1	Hepeviridae/ Hepevirus	974-1,184	AAA45734
Hepatitis E virus Ct1	HEV-4	Hepeviridae/ Hepevirus	988-1,198	Q9IVZ9
Avian hepatitis E virus	aHEV-1	Hepeviridae/ -	817-1,026	CAQ16027
Beet yellows virus	BYV	Closteroviridae/ Closterovirus	2,248-2,516	AAC25115
Mint virus 1	MV1	Closteroviridae/ Closterovirus	2,135-2,397	YP_224090
Plum bark necrosis stem pitting-associated virus	PBNSTaV	Closteroviridae/ Ampelovirus	2,046-2,309	YP_001552323
Little cherry virus 2	LChV-2	Closteroviridae/ Ampelovirus	1,344-1,606	NP_891562
